# Towards Scalable Strain Gauge-Based Joint Torque Sensors

**DOI:** 10.3390/s17081905

**Published:** 2017-08-18

**Authors:** Hamza Khan, Mariapaola D’Imperio, Ferdinando Cannella, Darwin G. Caldwell, Alfred Cuschieri, Claudio Semini

**Affiliations:** 1 The Institute for Medical Science and Technology, University of Dundee, Dundee DD1 4HN, UK; a.cuschieri@dundee.ac.uk; 2 Department of Advanced Robotics, Istituto Italiano di Tecnologia (IIT), 16163 Genova, Italy; mariapaola.dimperio@iit.it (M.D.); Ferdinando.cannella@iit.it (F.C.); darwin.caldwell@iit.it (D.G.C.); Claudio.semini@iit.it (C.S.)

**Keywords:** torque sensor, strain gauges sensor, joint torque control

## Abstract

During recent decades, strain gauge-based joint torque sensors have been commonly used to provide high-fidelity torque measurements in robotics. Although measurement of joint torque/force is often required in engineering research and development, the gluing and wiring of strain gauges used as torque sensors pose difficulties during integration within the restricted space available in small joints. The problem is compounded by the need for a scalable geometric design to measure joint torque. In this communication, we describe a novel design of a strain gauge-based mono-axial torque sensor referred to as *square-cut torque sensor (SCTS)*, the significant features of which are high degree of linearity, symmetry, and high scalability in terms of both size and measuring range. Most importantly, SCTS provides easy access for gluing and wiring of the strain gauges on sensor surface despite the limited available space. We demonstrated that the SCTS was better in terms of symmetry (clockwise and counterclockwise rotation) and more linear. These capabilities have been shown through finite element modeling (ANSYS) confirmed by observed data obtained by load testing experiments. The high performance of SCTS was confirmed by studies involving changes in size, material and/or wings width and thickness. Finally, we demonstrated that the SCTS can be successfully implementation inside the hip joints of miniaturized hydraulically actuated quadruped robot-*MiniHyQ*. This communication is based on work presented at the 18th International Conference on Climbing and Walking Robots (CLAWAR).

## 1. Introduction

In this paper, we focused on strain gauges-based joint torque sensors with the aim of exploiting their mechanical robustness [1] and scalability. It is based on work presented at the 18th International Conference CLAWAR [2]. Despite decades of reported studies on various strain gauges-based sensors for measuring joint torque over the years [3,4,5,6,7,8,9,10,11,12,13,14,15,16,17,18,19], their basic design has not improved significantly and most have restricted applicability in view of the strict requirements regarding linearity and symmetrical behavior to cope with different scales [20] necessitated by specific applications. The reason for this issue is well reported in literature, which clearly documented a trade-off between two factors i.e., sensitivity and torsional stiffness. The torsional stiffness is sacrificed at the expense of sensitivity, and vice versa. There is a third consideration that affects the design of a torque sensor. This relates to the means for securing fixation of the strain gauges on the torque sensor as this has an impact on both the shape and achievable performance.

This article’s focal point is designing and optimization of single axis torque sensor. The goal was to design a simple geometry that allows ease of bonding of the strain gauges at different physical sizes, while keeping the same level of performance in terms of high degree of linearity, symmetry, and high scalability in terms of both size and measuring range. The proposed sensor ((square-cut torque sensor (SCTS)) is born as a single axis; however, its geometry can be improved to measure multiple axes torques-forces by bonding additional strain gauges to each beam similar to multi-axes torque-force sensors [21,22,23,24] or the commercial sensors (such as Industrial Automation multi-axis force / torque (ATI) sensor), which measure the tangential forces along *x*-, *y*-, and *z*-axes as well as the moments about *x*-, *y*-, and *z*-axes simultaneously. However, SCTS measures the single axis joint torque and installs directly at joints, unlike other solutions that were installed into a mechanical power transmissions to measure in-line torque [25].

**Figure 1:**
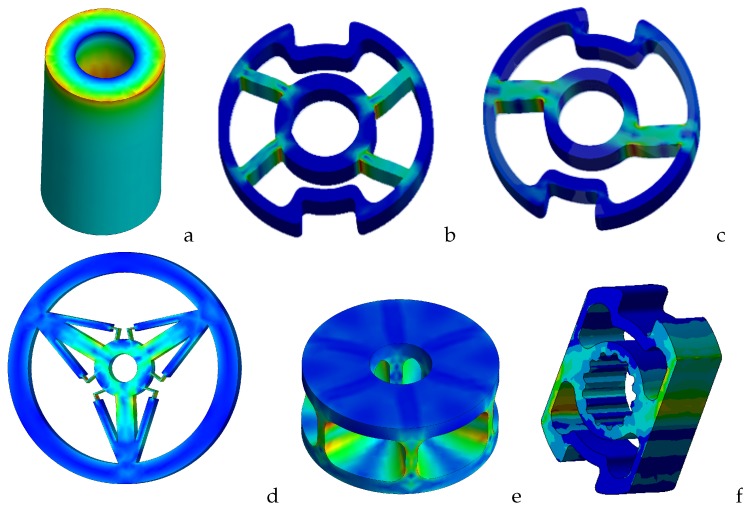
Various torque sensor geometries (**a**) hollow cylinder [3,4,26]; (**b**) hub-sprocket four spokes [5,6,27,28,29,30]; (**c**) hub-sprocket two spokes; (**d**) hub-sprocket with 4-bar linkage [9]; (**e**) hollow hexaform [31]; (**f**) new proposed design of Square-Cut Torque Sensor(SCTS), proposed in this communication.

The most common commercially available single axis torque sensors geometries are solid circular shafts, hollow circular shafts, cruciforms, hollow cruciforms, solid square, and hollow tubes with flats. The solid square offers advantages over the solid circular design; especially at high loading applications, it offers high bending strength and ease of application of strain gauges. Torque sensors with low loading applications are usually of the hollow cruciform type. The hollow cruciform structure produces high stress at low levels of torque, yet has good bending strength. These commercially available sensors, aside from being expensive, are hard to integrate into lightweight robot joints with restricted space. On the other hand, in-house custom made sensors often exhibit asymmetric behavior with respect to rotational direction and poor linearity, especially when miniaturized for direct-drive applications. The SCTS solution proposed by the present study overcomes all these issues because of its square-cut design, which ensures linearity, symmetry, high sensitivity and easy strain gauge positioning. Some of the reported solutions to resolve these issues are shown in Figure 1. The hollow cylinder torque sensor (Figure 1a), which has the simplest structure, is used extensively in many applications [3,4,26] despite its significant limitation concerning its geometry, rendering it intrinsically ultra-sensitive to non-torsional components [3]. The Hub-sprocket with four spokes (Figure 1b) is not sensitive to non-torsional components [5,6,27,28,29,30] compared with the hollow cylinder type, but it is difficult to position the strain gauges because of the compact geometry. Furthermore, it exhibits high stiffness and low sensitivity. An upgraded version (Figure 1c) known as the Hub-sprocket with two spokes has a bigger area for the positioning of the strain gauges in addition to a symmetric output. However, it does not detect peak stress, since the gauges are located at the midpoint of the beam that connects the inner with the outer cylinder, where only minimal deformations are possible. In the third version of the Hub-sprocket, the connection beams are substituted with 4-bar linkages. This design exhibits high sensitivity without sacrificing torsional stiffness because these are effectively decoupled: the stiffness being related to the spokes support portion, whereas the sensitivity is related to the 4-bar linkage sensing portion [9]. However, this complex geometry is hard to manufacture especially when miniaturized, which is often needed. The hollow hexaform solution proposed by Aghili [31] (Figure 1e) is both compact and stiff due to its high number of wing pairs. However, the reduction in size of the hollow hexaform design makes it difficult to access the surface gluing site for the strain gauges. The solution proposed (SCTS) in this communication (Figure 1f) has a square cut geometry, thereby exhibiting high degree of linearity, symmetry and scalability (both dimensional and measuring range). The SCTS uses silicon based strain gauges, and their big advantage is a very high gauge factor of about ±130, allowing measurement of small strain—for example, 0.01 microstrain [32].

**Figure 2:**
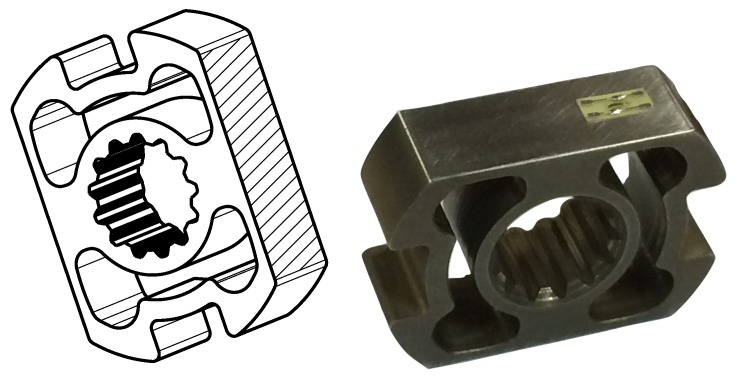
New *square-cut* joint torque sensor. (**left**) drawing highlighting the smooth surfaces where strain gauges can be glued on both wings; (**right**) a picture of the sensor.

Furthermore, it facilitates gluing and wiring of the strain gauges by virtue of its geometry, which allows direct access to the mounting surfaces, even in restricted areas, as can be seen in Figure 2. All of the aforementioned characteristics were studied initially by finite element modelling (ANSYS) and then tested by observational data obtained by experimental studies. The SCTS was then successfully installed in the hip joint of the MiniHyQ robot (Istituto Italiano di Tecnologia (IIT), Genoa, Italy) [33].

## 2. Torque Sensor Design

**Figure 3:**
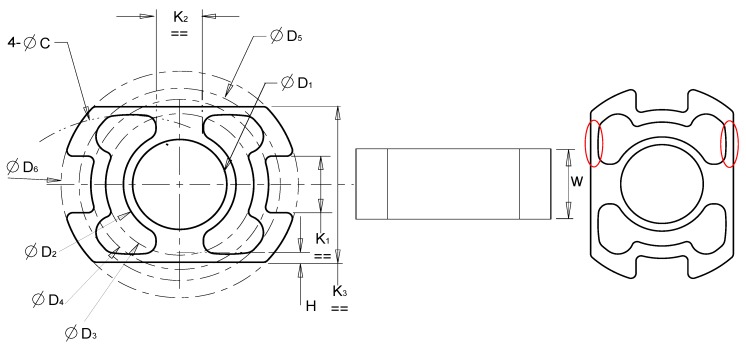
A drawing of easily customizable and compact strain gauge based *square-cut* joint torque sensor; (**left**) Computer-Aided Design (CAD) drawing; (**center**) side view of sensor; (**right**) strain gauges’ positions.

The SCTS is based on a novel square-cut design, which embodies all of the desired attributes needed for joint torque sensing, i.e., high degree of linearity, symmetry, scalability and easy mounting of strain gauges. The design parameters of the sensor geometry are shown and defined in Figure 3. The SCTS has two twin-wings that are stretched or compressed depending on the torque clockwise/counterclockwise rotation. The outer and inner diameter of the sensor ($D_{6}$, $D_{2}$, respectively) can be seen in Figure 3. The thickness and width of the two wings are labelled H and W, respectively, in the Figure 3 (Left,Center). The wings are curved, C, outlining a hollow cylindrical space to avoid unwanted buckling. The Parameters $K_{1}$ and $D_{4}$ define keyhole locking of sensor with the outer (driven) link. $K_{2}$ defines the symmetric separation distance between each side of the twin-wings. The strain-gauges are glued on to the respective outer surface of wings (encircled in red, Figure 3 (right)). They are electrically connected via half-bridge in order to maximize the signal and provide temperature compensation.

## 3. Analytical Model

**Figure 4:**
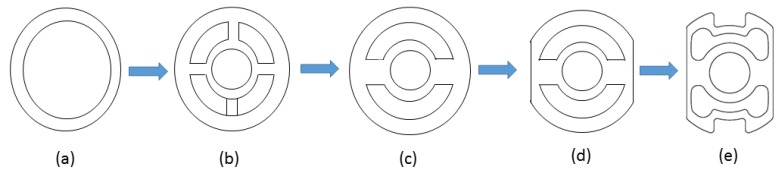
Evolution SCTS geometry which started from (**a**) hollow shaft; (**b**) hub-sprocket four spoke; (**c**) hub-sprocket two spokes; (**d**) square-cut hub-sprocket with two spokes to (**e**) optimized SCTS.

SCTS geometry evolution (in Figure 4) is started by considering a hollow shaft shape, and then adding an internal smaller hollow circular section, to be used as a connection for the motor axle, connected to the external one by means of four wings. This choice, as aforementioned, was abandoned because the wings were too flexible so that the higher strains were achieved on their surface that was smaller and uncomfortable for the strain gauges’ gluing. Thus, two wings were cut while the section of the remaining two was increased in order to transfer the higher strain values on the external surface of the sensor. This one was designed flat to have a wider and comfortable surface for the strain gauges’ gluing. The remaining surfaces were shaped instead in agreement with the requirement of the joint design.

**Figure 5:**
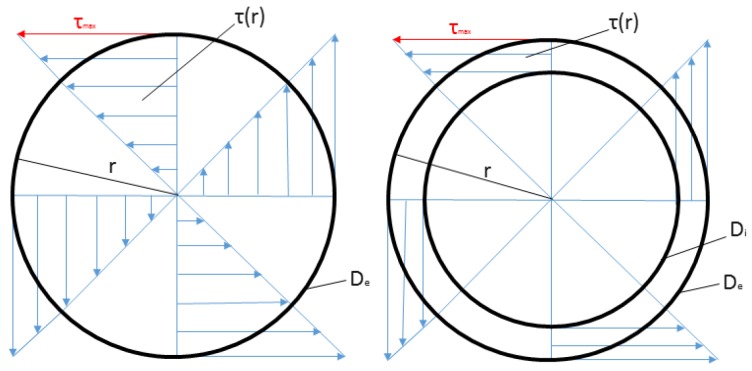
The shearing stress τ distribution in the plane of cross section for (**right**) solid shaft; (**left**) hollow shaft.

Torsion in solid shaft creates shearing stress τ, which varies directly as the distance ‘r’ from the axis of the shaft. The stress distribution in the plane of cross section can be seen in Figure 5 (left), which creates the complementary shearing stresses in an axial plane. The highest shear stress occurs on the surface of the shaft. When it is subjected to applied torque T, the stresses flow is given by $\tau =\frac{Tr}{I}$, where the radius r is maximum, $I=\frac{\pi {D_{e}}^{4}}{32}$ is the second moment of area and $D_{e}$ is the section diameter. However, the SCTS geometry was achieved starting from considering the analytical solution of torsion in a hallow shaft having closed cross section with thin walls, as shown in Figure 5 (right).

In the case of a hollow circular section, we have

(1) $$\tau_{max}=\frac{\left(16T\right)}{\left(\pi \left({D_{e}}^{3}-{D_{i}}^{3}\right)\right)},$$

where $D_{e}$ and $D_{i}$ are the external and the internal diameter, respectively. As consequence, the strains are given by

(2) $$\epsilon =\frac{\left(32TL\right)}{\left(\pi G\left({D_{e}}^{4}-{D_{i}}^{4}\right)\right)},$$

where L is the beam length and G is the shear modulus, which strictly depends on the material properties. As is it possible to notice in Equation (2), the strains’ values depends directly from both the value of the torque applied and the beam length, while, inversely, they depend on the material properties and geometry. Both stresses and strains are higher on the external surface of the beam that, for these reasons, became eligible for gluing the strain gauges.

**Figure 6:**
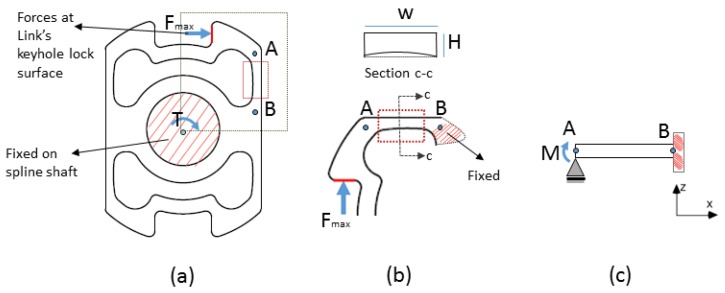
Simplified SCTS analytical structural model; (**a**) SCTS where the square box highlights a single wing; (**b**) close-up view of SCTS’s wing; (**c**) wing represented by cantilever beam.

We simplified SCTS analytical model by considering its single wing as it is highlighted in Figure 6a, where force $F_{max}$ was exerted by keyhole lock surface on SCTS (marked with red solid) and the second moment of inertia create a bending moment M at point A. Considering, SCTS’s wing geometry for Point A to Point B in Figure 6b can be represented by simple rectangular beam in Figure 6c. The uniaxial normal strain at this wing can be calculated by using its dimensions, the moment of inertia and position of the neutral axis. According to beam theory, a bending moment M ( M = F × d, where F is defined as a function of applied Torque T and design variables (see Figure 3), $F=\frac{T}{\frac{\left(K_{3}-H\right)}{2}}$ and $d=D_{4}-\frac{K_{2}}{2}$ ) at point A causes a uniaxial normal stress, $\sigma_{x}$, given by Equation (3)

(3) $$\sigma_{x}=\frac{3\frac{T}{\frac{\left(K_{3}-H\right)}{2}}\left(\frac{D_{4}}{2}-\frac{K_{2}}{2}\right)y}{\left(WH^{3}\right)},$$

where *y* is for distance from the neutral axis, SCTS’s wing width *W* and thickness *H*. The uniaxial normal strain $\varepsilon_{x}$ on each wing can be predicted by

(4) $$\varepsilon_{x}=\frac{\sigma_{x}}{E}=\frac{3\frac{T}{\frac{\left(K_{3}-H\right)}{2}}\left(\frac{D_{4}}{2}-\frac{K_{2}}{2}\right)y}{E(WH^{3})}=\frac{3T(D_{4}-K_{2})y}{E\left(WH^{3}\right)\left(K_{3}-H\right)},$$

where the elastic modulus, E, of the material. Due to complex geometry of SCTS, the final shape of SCTS is obtained by using finite element analysis (ANSYS simulation), and this is discussed in the next section.

## 4. Simulations and Analysis

Finite element analysis was used to obtain the final shape and to envisage the behavior of the torque sensors, and for sensitivity analysis with respect to four parameters: material, size, wing width *W* and thickness *H*. The simulations presented in this section demonstrate that it is possible to modify the performance of the torque sensor in terms of measurement scale and sensitivity without affecting its linearity and symmetry.

**Figure 7:**
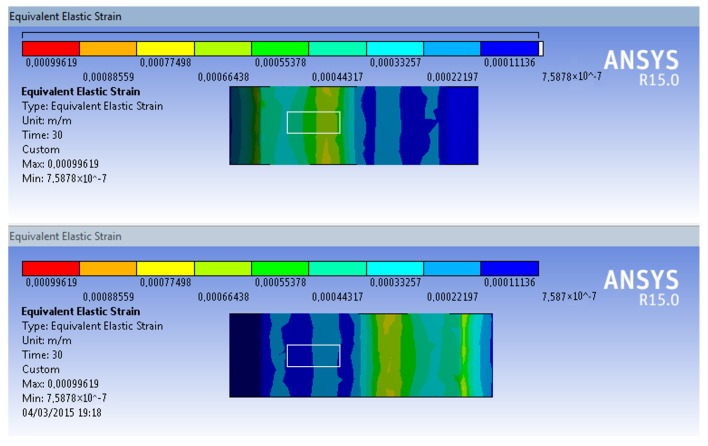
Simulated strain with clockwise applied torque at maximum value (60 Nm), where the white outlined box indicates the strain-gauge placement location; (**top**) upper surfaces; (**bottom**) lower surface.

**Figure 8:**
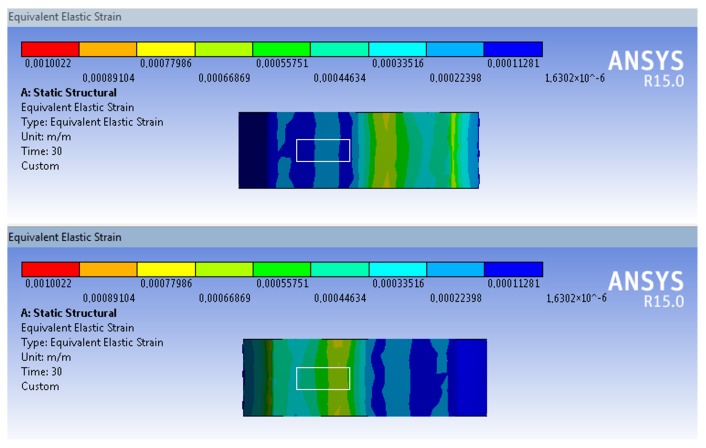
Simulated strain with counter-clockwise applied torque at maximum value (60 Nm) where the white outlined box indicates the strain-gauge placement location: (**top**) upper surfaces; (**bottom**) lower surface.

The SCTS structure, moreover, avoids residual differences between clockwise and counter-clockwise applied (due to machining, geometrical tolerances and material properties, etc.), thereby ensuring that the behavior remains symmetric, as shown in simulated strain in the Figure 7 and Figure 8. The strain-gauges are placed at external flat surfaces, and this is indicated by a white outlined box at the upper and lower surfaces of left side wings. Another significant improvement resulting from the twin-wing design is that of empowering SCTS with exhibiting a linear behavior together with high strain because the wings are only stressed within minor displacements, ensuring linear strain [34], but it also facilitates attachment of the strain-gauges to the maximal strain point. In addition, the gluing site for the strain gauges was specifically located on the outer flat surface of SCTS, and this is shown by a white outlined box in Figure 8.

### 4.1. Numerical Model

Numerical simulations were performed to investigate the parameters influencing the torque sensor behavior, e.g., material used, scale, wing width and thickness. Table 1 summarizes the simulation plan and parameters investigated. All of the ratios in Table 1 refer to the actual dimensions of the physical prototype (see Section 4). Three materials were studied Yield strength: Steel 39NiCrMo3 835 MPa; Titanium alloy Ti-64 510 MPa; 7075 Aluminium alloy (Ergal) 435 MPa; : Steel 39NiCrMo3, Titanium alloy Ti-64 and 7075 Aluminium alloy (Ergal).

**Figure 9:**
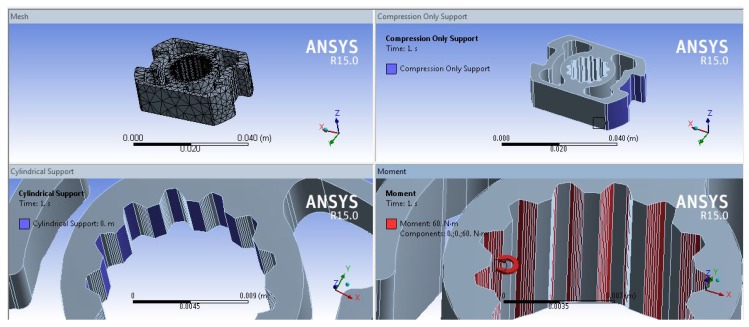
Finite element model: (**top left**) the mesh; (**top right**) the contact constraint between the sensor and the case; (**bottom left**) the fix constraint; (**bottom right**) the applied torque.

The analysis showed that Titanium and Aluminium alloy behaved weakly when compared to the steel alloy 39NiCr3Mo due to their mechanical properties. The torque sensor scale ratios studied were 1:0.75 and 1:1.25, respectively. The wing width *W* and thickness *H* were each investigated using high and low ratios (Table 1). All of the simulations were carried out with ANSYS r15 program (ANSYS, Inc., Canonsburg, PA, USA), using a quadratic element mesh with six degrees of freedom for each node, suitable both for linear and for nonlinear applications (SOLID 189, ANSYS user manual), shown in Figure 9. The constraint and the load applied reproduced the experimental tests conditions (see Section 5).

**Figure 10:**
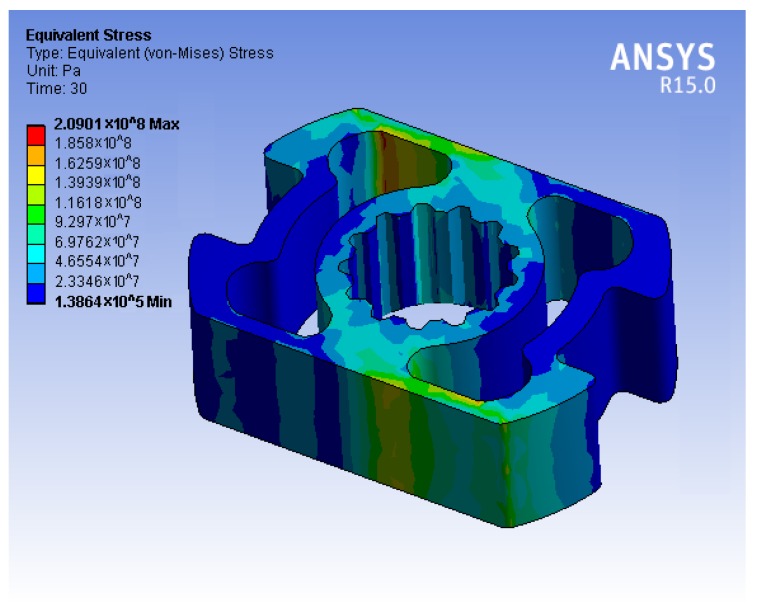
The simulated von Mises Stress at maximum load (60 Nm) demonstrates that the maximum stress value of the torque sensor is about 25% of the yield point of the steel alloy 39NiCr3M.

At the start of the simulation, the stress was checked to ensure that the torque sensor was well within the yield strength point for different materials, before the parameters were investigated. It can be seen in Figure 10 for the steel alloy 39NiCr3Mo. The following analysis indicated that SCTS exhibited a linear and symmetric behaviour, the details and recording of which are reported in the remainder of this section.

### 4.2. Effects of Material

**Figure 11:**
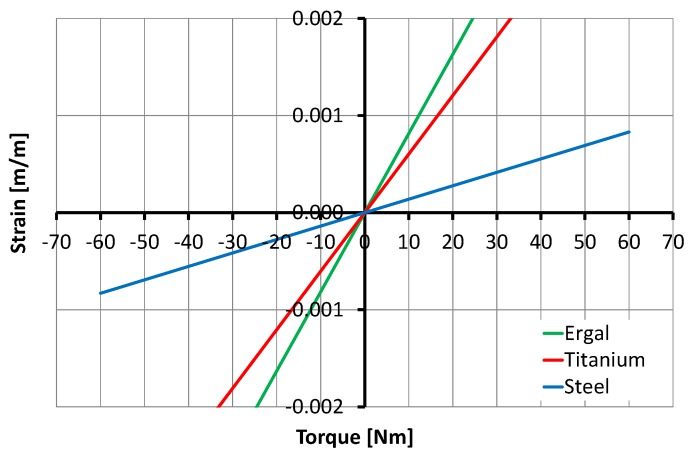
Relationship between the torque and the strain for three material studies.

Three materials were investigated to determine their effect on strain while considering fixed initial sizing of SCTS and maximum applied torque. The 7075 Aluminium (Ergal) was found to be very stressed when reaching

the yield point at 25 Nm of torque and the stress on the titanium averaged 33 Nm, indicating that both materials were unsuitable for the intended load (60 Nm) for the same physical dimensions of SCTS. As shown in Figure 11, the only material with the strength to cope with the desired torque load is a steel alloy 39NiCrMo3 for the same physical dimensions of SCTS.

There is a difference between the Aluminium alloy(Ergal), Titanium alloy Ti-64 and Steel 39NiCrMo3 material stiffness ratio (see in note Yield strength: Steel 39NiCrMo3 835 MPa; Titanium alloy Ti-64 510 MPa; 7075 Aluminium alloy (Ergal) 435 MPa) and the strain values (as shown in the Figure 11) ratio. This is given by the torsional moment that generated flexural moment M = F × d on the wing ends (as shown in the Figure 6). There is a geometrical nonlinearity in the Equation (4), depending on $\frac{\left(K_{3}-H\right)}{2}$ and *y* that change with respect to the applied torque T, which causes strains in the Ergal that seem to be larger than steel [35,36].

### 4.3. Effects of Scaling

The overall size of the sensor varied incrementally (increased and decreased by 25%).

**Figure 12:**
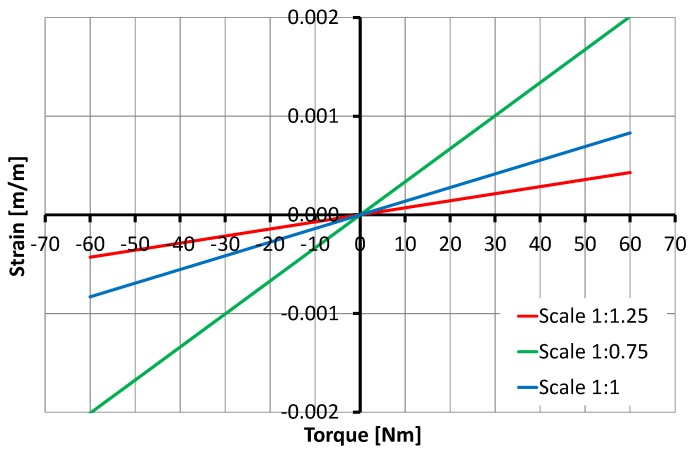
Relationship between torque and the strain with size: variations of 25% influence torque sensor behavior.

The design parameters for this simulation are $D_{1}=15$ to 25 mm, $D_{2}=19.5$ to 33.5 mm, $D_{3}=22.5$ to 37.5 mm, $D_{4}=24$ to 40 mm, $D_{5}=27$ to 45 mm, $D_{6}=30$ to 50 mm, $K_{1}=9$ to 15 mm, $K_{2}=11.25$ to 18.75 mm, W=11.25 to 18.75 mm and H=3 to 5 mm. The results demonstrate that size has a significant direct relation on strain, the strain being doubled or halved, as shown in Figure 12.

### 4.4. Effects of Wing Width and Thickness

**Figure 13:**
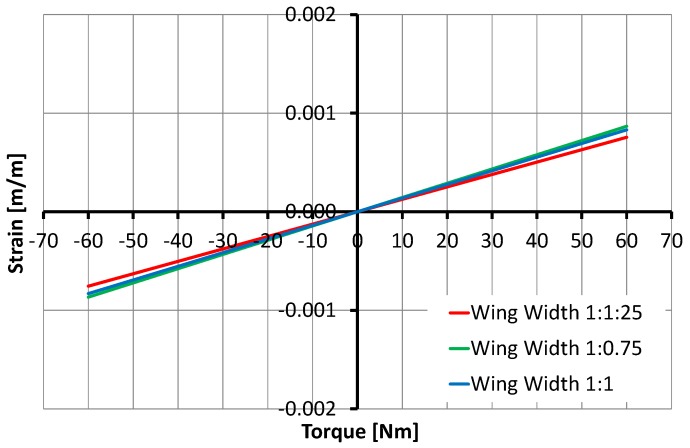
Relationship between torque and the strain depending on the wing width showing that 25% variation of width has negligible effects.

**Figure 14:**
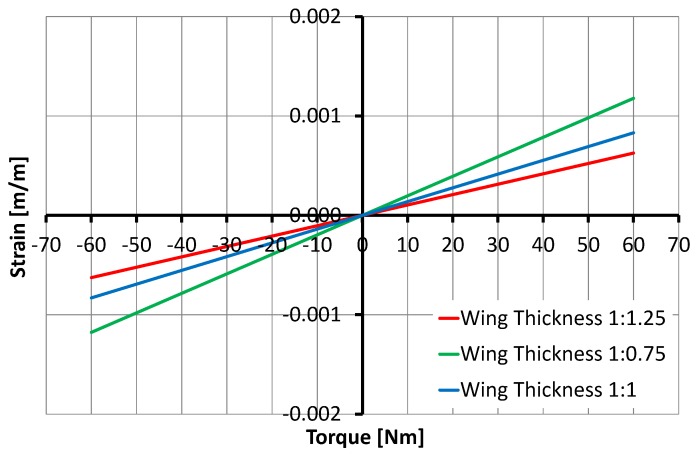
Relationship between the torque and the strain depending on the wing thickness parameter: the 25% of variation influences torque sensor behavior. The reduction increases more than 40% the strain and the increment of the thickness reduces it by 25%. This means that the width reduction has more influence than the width increment.

The variation in SCTS’s wing width *W* and thickness *H* significantly influenced the strain rate. In particular, the increment of 1.25 of width is less sensitive than 1.25 of thickness, and this was expected according to the applied design rules [37], as shown in Figure 13 and Figure 14.

### 4.5. Effects of $D_{1}$ $D_{6}$ $K_{1}$ and $K_{2}$

**Figure 15:**
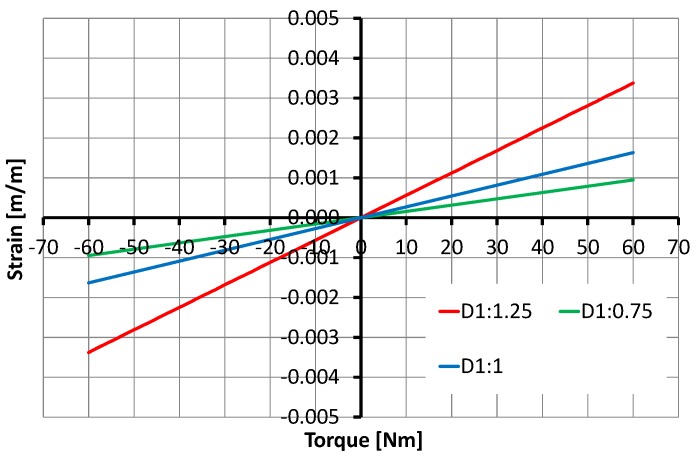
Relationship between the torque and the strain depending on the variation of SCTS’s inner shaft diameter $D_{1}$.

**Figure 16:**
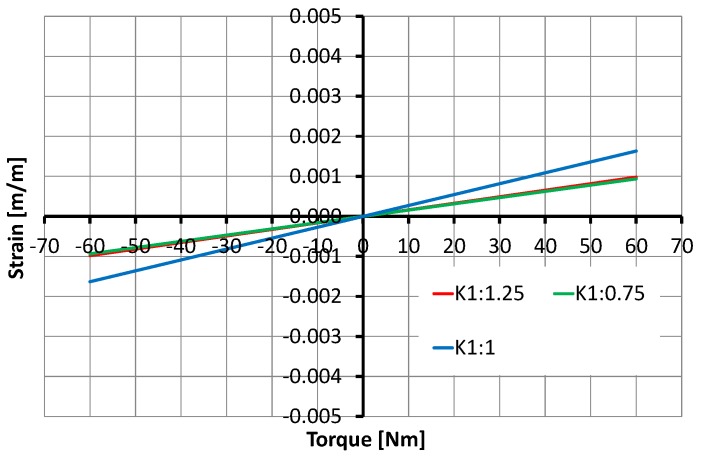
Relationship between the torque and the strain depending on design parameter $K_{1}$: the 25% of variation influences torque sensor behavior.

**Figure 17:**
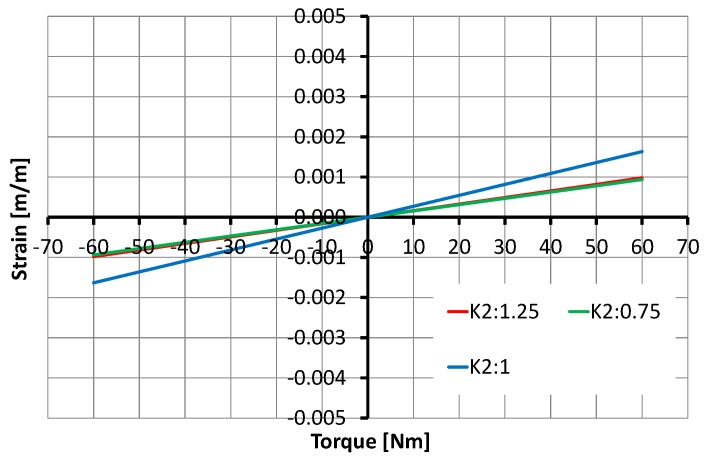
Relationship between the torque and the strain depending on design parameter $K_{2}$: the 25% of variation influences torque sensor behavior.

The selection of SCTS’s inner shaft diameter $D_{1}$ and outer diameter $D_{6}$ is mainly constrained by the available joint space where SCTS need to be fitted. By fixing its outer diameter $D_{6}$, the variation in SCTS’s inner shaft $D_{1}$ can be seen in Figure 15. Once $D_{1}$ and $D_{6}$ are defined, then the rest of the design parameters follow them. However, the increment or decrement in $K_{1}$ and $K_{2}$ by 25% exhibits similar behavior and it can be seen in Figure 16 and Figure 17.

## 5. Experimental Results

**Figure 18:**
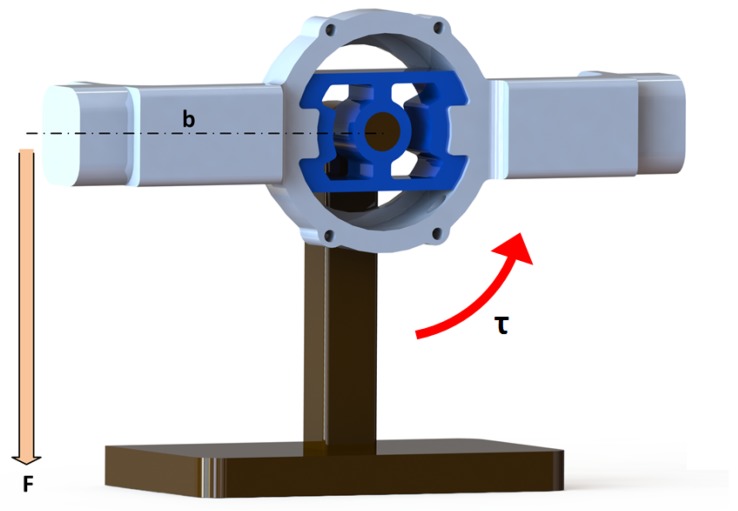
Experimental set up layout, the clockwise/counter clockwise torque τ is generated about sensor by inserting force F at a perpendicular distance b at the right or left end of the beam.

Based on FEM simulation data, SCTS was machined of the steel alloy 39NiCr3Mo. The half bridge strain gauges were easily glued on both sides of the sensor to maximize the signal and provide temperature compensation. An experimental test rig layout is shown in Figure 18, where the relevant end of beam is loaded with weight at fixed lever-arm *b* in order to apply clockwise/counter-clockwise torque on the SCTS.

**Figure 19:**
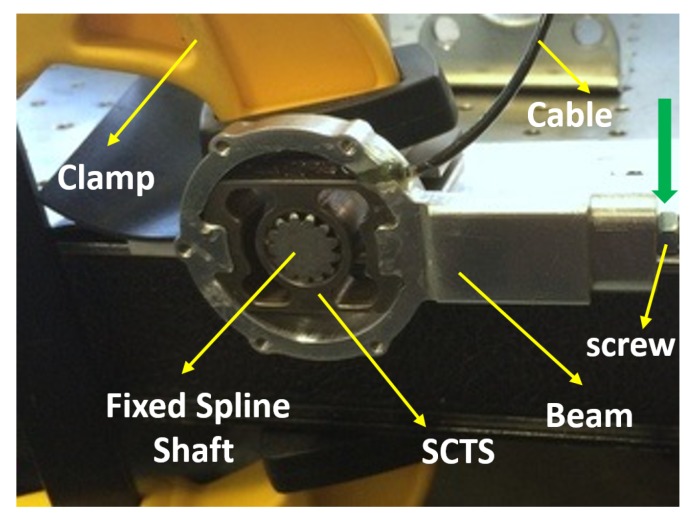
This experimental setup hardware mainly consists of three parts i.e., the spline shaft, the SCTS and beam. The spline shaft is fixed on the table and torque sensor inner shell is locked on it with zero-mechanical play. The beam that is locked on outer key locks of sensor and load is applied at its end in order to generate the torque. The attachment screw is used for the extension of the beam to increase its lever arm b.

The experimental setup for testing the SCTS is shown in Figure 19, and the torque is generated from placing a weight on the beam end. The applied torque is defined as τ=m×g×b, where g is the gravity acceleration. It is varied by changing weight ’m’ applied on beam. This torque sensor is designed measuring a maximum torque of 60 Nm. Its design parameters are $D_{1}=$ 20 mm, $D_{2}=$ 26 mm, $D_{3}=$ 30 mm, $D_{4}=$ 32 mm, $D_{5}=$ 36 mm, $D_{6}=$ 40 mm, $K_{1}=$ 12 mm, $K_{2}=$ 15 mm, W= 15 mm and H= 3 mm (see Figure 3). The safety factor for SCTS is 3.96, and it is computed using ANSYS simulation working stress results at intended load and material yield stress.

An in-house constructed signal processing board was used to amplify the output of SCTS analog signal and its conversion to 18-bit digital signal.

### 5.1. Validation

**Figure 20:**
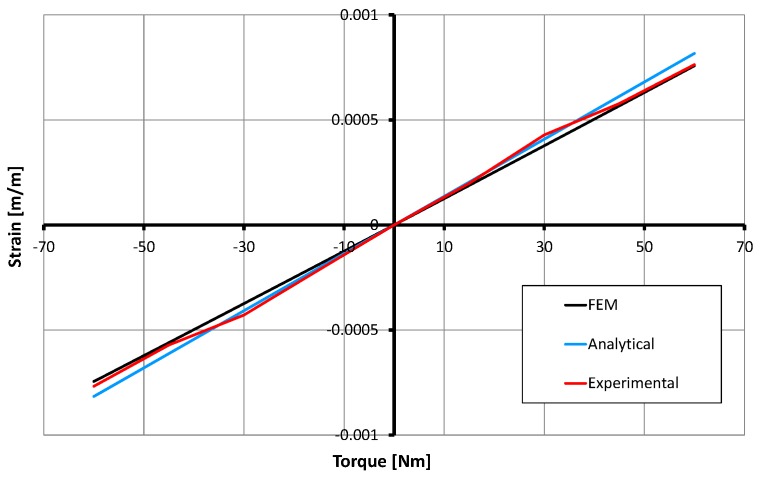
The comparison between finite element method (FEM) simulation, analytical estimation (see Equation (4)) and direct experimental results.

The experimental data are compared with the FEM simulation and analytical estimation data. It is shown in Figure 20 confirming the reliability of the SCTS design in terms of its linear and symmetric output. Obvious discrepancy between experimental and FEM results at the torque value of ± 30 Nm can be seen. It is due to the nonlinearity of the strain gauges and bonding glue under the strain gauges. Moreover, this small difference between the FEM simulation and direct experiments will be used for future refinements of the SCTS torque sensor to further enhance its *accuracy* and *reliability*.

**Figure 21:**
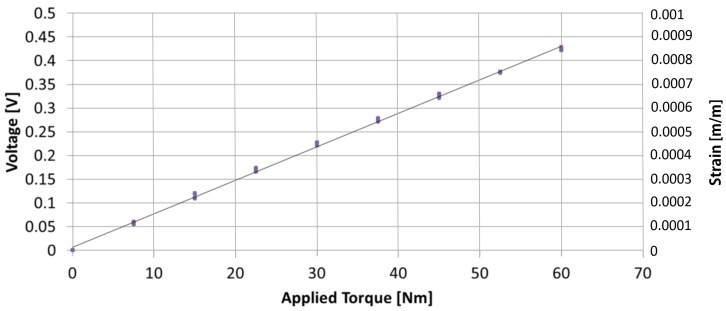
Calibration and regression curve.

According to the calibration curve (it can be seen in Figure 21), the following SCTS measurement characteristics are determined [38,39]: sensitivity: 141.3 mV/Nm, offset: 6.7 mV, linearity: 0.23% of F.S, full scale: 60 Nm and accuracy: 0.5% and noise and signal to noise: 3.7%.

## 6. Conclusions

**Figure 22:**
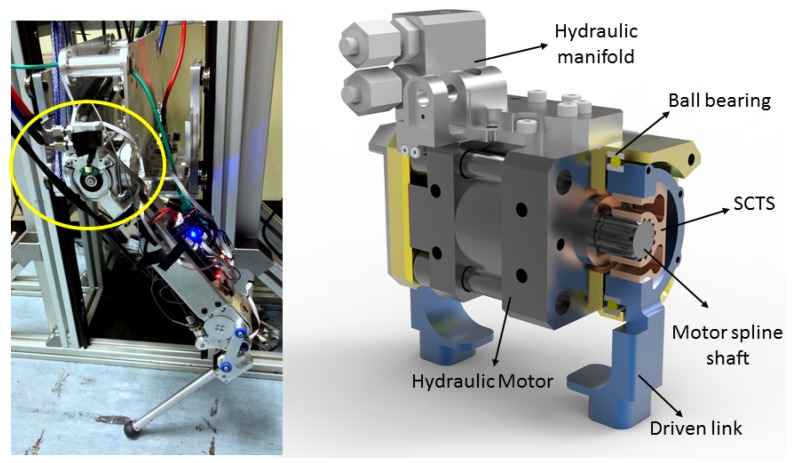
(**left**) Picture of MiniHyQ single leg where hip joint encircled in yellow; (**right**) CAD model of MiniHyQ hip joint’s hydraulic motor, where SCTS is fitted into motor spline shaft and its outer keyholes are locked with lower driven link.

This paper presented details of a novel SCTS design that is easily customizable for compact joint torque measurements. SCTS was also successfully integrated into the hip joint of each *miniaturized hydraulically actuated quadruped robot-MiniHyQ* [33] leg, and it can be seen in Figure 22 (left). The MiniHyQ hip joint computer-aided design (CAD) is shown in Figure 22 (right), where the SCTS center is locked on the hydraulic rotary motor’s spline shaft, and its outer keyholes are locked with a driven link. In this application, SCTS provides efficient and reliable hip joint torque sensing up to a maximum of 60 Nm.

The novel joint torque sensor, apart from its simple design, exhibits linear and symmetric output coupled with high sensitivity in both clockwise and counter clockwise directions. These attributes are underpinned by the robust nature of the sensor structure, easy access for strain-gauge gluing/fixing and the half bridge electric connection. SCTS is instantaneously capable of reporting the amount of torque applied with a sensitivity of 141.3 mV/Nm. It meets a range of requirements depending on the intended applications. In practice, it is possible to modify torque sensor performance in terms of measurement range and sensitivity without adversely affecting its linearity and symmetry, simply by altering overall size, material type and/or wings width and thickness. The study showed reasonable correlation between simulation (FEM) predicted and observed experimental data. The initial un-strained bridge measurement (output signal) was accounted for to avoid restriction on the obtained resolution and nullified the offset of the initial voltage. We used 18-bit analogue-to-digital converter to provide 3.4 μV of resolution, which ensured the reading of the desired signal. However, the effects of design parameters on SCTS bandwidth and dynamic measurements, including the resolution at higher frequencies, will be reported in future work. Hence, future research is also envisaged to optimize the structure in order to improve strain rate. In these future studies, several design parameters will be explored as outlined in Figure 3. Additionally, the application of SCTS in different scale rotary joints will be evaluated. 

## Figures and Tables

**Table 1:** Overview of preformed numerical simulations.

Case	Nodes	Elements	Degree of freedom (DOF) for Each Simulation	Material	Scale	W	H
0	262740	13137	1576440	39NiCrMo3	1:1	1:1	1:1
1	262740	13137	1576440	7075 Aluminium	1:1	1:1	1:1
2	262740	13137	1576440	Ti64 Titanium	1:1	1:1	1:1
3	262240	13112	1573440	39NiCrMo3	1:1.25	1:1	1:1
4	262380	13119	1574280	39NiCrMo3	1:0.75	1:1	1:1
5	288100	14405	1728600	39NiCrMo3	1:1	1:1.25	1:1
6	205860	10293	1235160	39NiCrMo3	1:1	1:0.75	1:1
7	271040	13552	1626240	39NiCrMo3	1:1	1:1	1:1.25
8	249840	12492	1499040	39NiCrMo3	1:1	1:1	1:0.75

## Abbreviations

The following abbreviations are used in this manuscript:

SCTS	Square-Cut Torque Sensor
FEM	Finite Element Method
FEA	Finite Element Analysis
DOF	Degree of Freedom
HyQ	Hydraulically actuated Quadruped robot
MiniHyQ	Miniaturized Hydraulically actuated Quadruped Robot
CAD	Computer-Aided Design
IIT	Istituto Italiano di Tecnologia
